# Empirical Evaluation of the Relative Range for Detecting Outliers

**DOI:** 10.3390/e27070731

**Published:** 2025-07-07

**Authors:** Dania Dallah, Hana Sulieman, Ayman Al Zaatreh, Firuz Kamalov

**Affiliations:** 1 Department of Mathematics and Statistics, American University of Sharjah, Sharjah 26666, United Arab Emirates; g00078476@alumni.aus.edu (D.D.); aalzaatreh@aus.edu (A.A.Z.); 2 School of Engineering, Applied Science and Technology, Canadian University Dubai, Dubai 14143, United Arab Emirates

**Keywords:** outlier detection, range distribution, order statistic, data analysis

## Abstract

Outlier detection plays a key role in data analysis by improving data quality, uncovering data entry errors, and spotting unusual patterns, such as fraudulent activities. Choosing the right detection method is essential, as some approaches may be too complex or ineffective depending on the data distribution. In this study, we explore a simple yet powerful approach using the range distribution to identify outliers in univariate data. We compare the effectiveness of two range statistics: we normalize the range by the standard deviation (σ) and the interquartile range (IQR) across different types of distributions, including normal, logistic, Laplace, and Weibull distributions, with varying sample sizes (*n*) and error rates (α). An evaluation of the range behavior across multiple distributions allows for the determination of threshold values for identifying potential outliers. Through extensive experimental work, the accuracy of both statistics in detecting outliers under various contamination strategies, sample sizes, and error rates (α=0.1,0.05,0.01) is investigated. The results demonstrate the flexibility of the proposed statistic, as it adapts well to different underlying distributions and maintains robust detection performance under a variety of conditions. Our findings underscore the value of an adaptive method for reliable anomaly detection in diverse data environments.

## 1. Introduction

In data analysis, ensuring the quality and reliability of data is important for making informed decisions and extracting meaningful insights [1]. However, datasets often contain irregularities known as outliers, which can significantly distort statistical analyses and mislead predictive models. An outlier is a data point that deviates substantially from other observations, either due to errors, natural variation, or rare events [2]. If not properly identified and handled, outliers can affect the results of studies, making outlier detection a critical task in data analysis.

Outlier detection, also known as anomaly detection, refers to the process of identifying data points that deviate significantly from the majority of the data. These points often “lie outside” the expected pattern or distribution, making them potential anomalies. Outliers can skew analyses, especially in fields such as fraud detection [3,4], medical diagnosis, network security, and scientific research. For instance, outlier detection can be used to uncover fraudulent transactions, diagnose potential health issues [5], and detect cyberattacks [6], and it can even lead to groundbreaking scientific discoveries. Identifying outliers is challenging due to the unique characteristics of each dataset. While some outliers may be the result of measurement errors or data corruption, others may represent valuable insights. Statistical methods can be used to flag these anomalies, but interpretation often requires domain expertise. Outliers can occur in both univariate and multivariate contexts, necessitating specialized techniques to detect and analyze them.

The role of outlier detection has become increasingly vital in today’s world, where diverse fields rely on accurate data analysis to make informed decisions. As digital data continue to expand in volume and complexity, the efficient detection of outliers is fundamental to managing unexpected patterns and extracting meaningful knowledge from vast datasets, ultimately reinforcing the dependability of data-driven systems across sectors.

Numerous outlier detection methods are found in the literature. The most widely used outlier method is the boxplot, which has been applied in various fields of study. Tukey’s boxplot is a graphical tool used to visualize the distribution of data, highlighting the median, quartiles, and potential outliers based on the interquartile range (IQR) [7]. An IQR defined by $Q_{3}-Q_{1}$ is used as a robust measure of statistical dispersion; $Q_{1}$ and $Q_{3}$ denote the first and third quartiles, respectively.

Tukey proposed identifying outliers as observations lying outside the interval $\left[Q_{1}-1.5\;\mathrm{IQR},\;Q_{3}+1.5\;\mathrm{IQR}\right]$. However, its effectiveness diminishes when dealing with skewed data distributions. One of the primary issues with Tukey’s boxplot is that it often constructs fences (thresholds for identifying outliers) that extend too far from the data on the compressed side of the distribution while declaring erroneous outliers on the extended side. To address this problem, Ref. [8] introduced an adjustment to Tukey’s technique, incorporating a robust measure of skewness known as “Medcouple.” However, this adjustment sometimes leads to the construction of fences that extend beyond the extremes of the data, rendering it ineffective in detecting outliers. Findings demonstrate that the modified technique’s fences are closer to the true 95 percent values compared to the adjusted boxplot, indicating its superiority in outlier detection, especially for skewed data. Another useful outlier detection method is Grubbs’ test, which is commonly used in various engineering fields, particularly in quality control and industrial engineering [9]. Grubbs developed a statistical test to determine whether a single outlier exists in a univariate dataset. For a sample of *n* observations ($x_{1},x_{2},...,x_{n}$) assumed to be normally distributed, with $x_{n}$ representing the most extreme observation, this test assesses the null hypothesis that the dataset contains no outliers, contrasting it with the alternative hypothesis that an outlier is present.

More recently, the authors of [10] proposed a boxplot-based outlier detection method that enhances the traditional IQR approach by modifying the fences with semi-interquartile ranges tailored to specific location-scale distributions, such as logistic and exponential distributions. By using distribution-specific constants for the fence boundaries, their approach accounts for the underlying shape of the data, which improves the accuracy of identifying outliers across skewed and symmetric distributions. This work demonstrates the importance of adjusting detection methods based on distributional properties to maintain a controlled error rate, particularly the “some-outside rate,” which measures the probability of falsely labeling a data point as an outlier within a given sample size.

One significant advancement in outlier detection can be found in [11]; this work proposed the standardized range statistic, which is a range of the observations relative to the standard deviation. This approach was a step forward, yet its applicability remains limited for skewed distributions. Building on these methods, Ref. [12] recently introduced the relative range statistic, designed to enhance the standardized range approach by offering a more adaptable measure for outlier detection. The statistic was initially applied to the normal and Weibull distributions [13,14], chosen to represent the symmetric and skewed cases, respectively. Through simulation studies, the statistic demonstrated advantages in accuracy and robustness over traditional methods, especially in its ability to accurately detect outliers without disproportionately flagging observations in skewed data. The promising results of this proposed technique in handling varied distributions suggest potential for further application across other location-scale families.

The current study seeks to examine the applicability of the statistic by extending it to additional distributions, namely, logistic and Laplace distributions, each characterized by unique location-scale properties and tail behaviors. Inspired by the work in [10], this paper examines how the relative range performs when applied to logistic and Laplace distributions. The goal is to evaluate the flexibility and robustness of the proposed statistic across a broader spectrum of distributions, providing a more comprehensive approach to outlier detection that can adapt to varying shapes and scale characteristics.

The remainder of this paper is organized as follows: Section 2 introduces the range test statistic used for outlier detection and empirically investigates its distributional characteristics using randomly generated samples from normal, logistic, Laplace, and Weibull distributions. Section 3 presents a methodology to assess the performance of the proposed range statistic in detecting outliers, with comparisons to the the performance of the standardized range introduced in [11]. Section 4 discusses the experimental results, demonstrating that the proposed statistic is more robust and effective than the standardized range. Finally, Section 5 provides some conclusions and insights for future work.

## 2. Methodology

In statistics, the range is a statistical measure that indicates how diverse the data values are in a dataset. It can be a useful tool for detecting outliers, where the existence of one extreme value at either end of the dataset results in an entirely different and inflated range. The range statistic was introduced in [11], primarily for identifying outliers in a sample from a normal distribution. The author proposed the following statistic:

(1) $$W=\frac{R}{\sigma },$$

where *R* is the sample range, and σ is the population standard deviation.

A table of percentage points of the range distribution was constructed for normally distributed data with different sample sizes up to n = 100. While *W* is a useful tool for detecting outliers, it has a significant drawback. As noted in [14], if σ is estimated externally (such as from the sample itself), *W* can become highly sensitive to outliers. This is because outliers can inflate the standard deviation, which may lead *W* to falsely identify outliers, even when their impact is minimal.

In an attempt to overcome this issue, a more robust measure of variation, namely, the interquartile range, is proposed here. In [14], the authors proposed standardizing the range, *R*, by the interquartile range, IQR. They defined the *K* statistic as

(2) $$K=\frac{R}{IQR}$$

Unlike the standard deviation, which takes all values into account and can be heavily influenced by extreme points, the IQR focuses on the middle 50 percent of the data. As a result, it provides a more stable summary of variability, especially in datasets that contain outliers. Using the IQR instead of the standard deviation allows for a more accurate representation of the data’s core structure, reducing the risk of distortion caused by unusually large or small values.

The authors called *K* the relative range because it adjusts the range of the entire dataset relative to the range of the middle half of the dataset. The authors first explored the distributional behavior of *K* and its performance in detecting anomalous observations by constructing the probability density function (PDF) and cumulative distribution function (CDF) using the concept of order statistics. Looking at the CDFs, they concluded that the analytical construction and interpretation of the probability behavior of *K* can be challenging. Therefore, the probability behavior of the relative range, *K*, was explored empirically through simulation experiments in order to shed some light on its probability distribution characteristics and establish some understanding of its performance. The simulation experiments in [13,14] were conducted using data generated from the normal and Weibull distributions for a fixed error rate of α=0.05. The resulting probability density functions (PDFs) for *W* and *K* exhibited consistent behavior across different sample sizes, with *K* showing a slightly greater rightward skewness than *W* for smaller sample sizes. Numerical simulations have shown that the *W* statistic is particularly sensitive in detecting outliers for n≤100, whereas the *K* statistic provides a more stable and precise identification of anomalous observations in that range. Interestingly, for sample sizes greater than 100, the performance of *W* improves and becomes comparable to that of *K*, which may explain why the generated tables in [11] were limited to n=100: beyond that point, the statistical advantage of *K* in detecting outliers becomes comparable to that of *W*.

In this work, we aim to explore the distributional behavior of *K* and its performance in detecting outliers, and we compare it to the performance of the standardized range, *W*. We first examine the probability behavior of the relative range, *K*, for various distributions. Unlike previous studies, which primarily focused on normal and Weibull distributions, this work extends the analysis to also include logistic and Laplace distributions. The behavior of *K* is explored empirically through simulation experiments in order to shed light on its probability distribution characteristics and to better understand its performance in detecting anomalous observations. Furthermore, while earlier work evaluated the range statistics at a single error rate, our study investigates the performance of both *W* and *K* across multiple error rates: α=0.1,0.05, and 0.01. An important advantage of this approach is that it leverages the underlying distributional characteristics of the data to inform the detection of outliers, providing an adaptable framework for anomaly identification.

### 2.1. Distributional Behavior of W and K from Different Distributions

We utilize the program RStudio version 4.4.1 as our programming tool to conduct a simulation experiment aimed at investigating the probability distribution of *W* and *K*. This experiment aims to explore how these statistics behave across various distributions and sample sizes, providing insights into their properties and potential applications.

#### 2.1.1. Normal Distribution

The simulation process involves random sampling from the standard normal distribution, with a location of 0 and a scale of 1. We generate multiple random samples, each of a specified size denoted as *n*, and we calculate the statistics *W* and *K* for each sample. We perform 10,000 simulations for each of the chosen sample sizes. To gain insights into their distributions, we visualize them by empirically plotting their distributions using the kernel density function.

**Figure 1:**
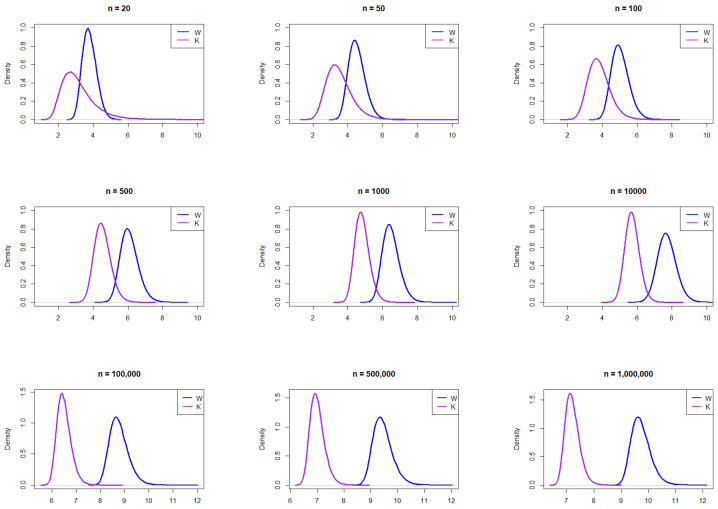
Empirical exploration of the distributions of *W* and *K* for n=20, 50, 100, 500, 1000, 10,000, 100,000, 500,000, and 1,000,000 from the normal distribution N(0,1).

Figure 1 illustrates the empirical PDFs of *W* and *K* from the normal distribution for various sample sizes. The PDFs for *W* and *K* exhibit consistent behavior across sample sizes. The relative range shows slightly more right skewness than *W*, indicating that *K* can take larger values for smaller sample sizes, making it more capable of capturing outlying values in bell-shaped curves than *W*. Beyond a sample size of 100, *W* exhibits right skewness and shifts further to the right of *K* assuming larger values.

#### 2.1.2. Logistic Distribution

To further explore how distributional differences influence the behavior of W and K, we extend our analysis to the logistic distribution. Following the same simulation procedure, we generate random samples from the logistic distribution, with a location of 0 and a scale of 1.

**Figure 2:**
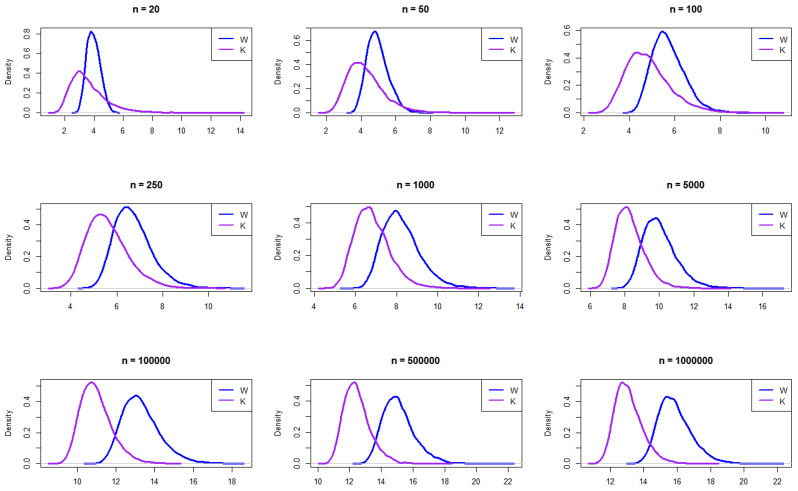
Empirical exploration of the distributions of *W* and *K* for n= 20, 50, 100, 250, 1000, 100,000, 500,000, and 1,000,000 from the logistic distribution.

The resulting empirical PDFs of *W* and *K* from the logisitic distribution for the different *n* values are plotted in Figure 2. The figure demonstrates that the PDFs for *W* and *K* generally align in behavior across sample sizes. Similar to the normal distribution, *K* exhibits more right skewness than *W*, particularly for smaller sample sizes. Beyond n=100, *W* shows a subtle shift to the right and becomes slightly skewed.

#### 2.1.3. Laplace Distribution

The Laplace distribution has heavier tails than both the normal and logistic distributions. Multiple random samples are generated from the Laplace distribution, with a location parameter of 0 and a scale parameter of 1, following the same simulation procedure.

**Figure 3:**
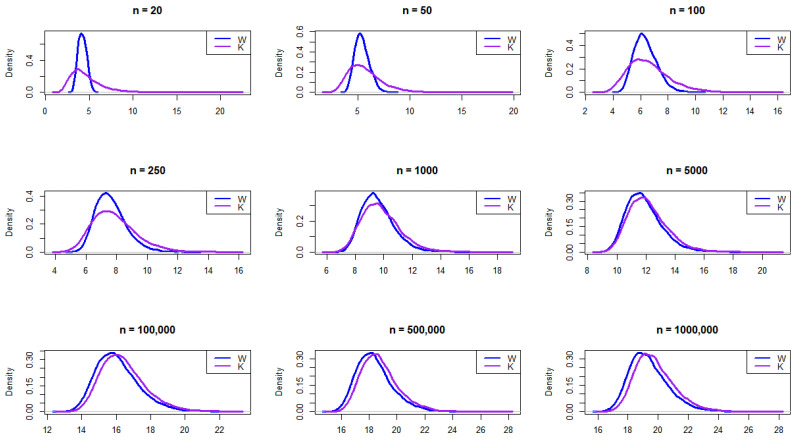
Empirical exploration of the distributions of *W* and *K* for n= 20, 50, 100, 250, 1000, 100,000, 500,000, and 1,000,000 from the Laplace distribution.

The resulting empirical PDFs of *W* and *K* for the different sample sizes from a Laplace distribution are plotted in Figure 3. The figure demonstrates that, overall, at smaller sample sizes (n=20, 50, and 100), the distributions exhibit noticeable differences, with *W* appearing to have a sharper peak and *K* showing a broader, lower curve. As the sample size increases, the densities of both distributions converge closer together, indicating greater similarity in shape. By n=100,000 and beyond, both distributions become almost identical, suggesting that, with large sample sizes, the distributions of *W* and *K* approach similar density functions. This trend implies that, while the distributions differ significantly at small sample sizes, asymptotically, they exhibit similar distributional characteristics.

#### 2.1.4. Weibull Distribution

Finally, we analyze the two-parameter Weibull distribution, which is defined by a shape parameter and a scale parameter. Unlike the previous distributions, the Weibull distribution exhibits skewness that is directly influenced by its shape parameter. This parameter is particularly important because it influences the form of the probability density function (PDF) and, as a result, the overall behavior of the distribution. Depending on its value, the Weibull distribution can resemble other well-known distributions. For instance, when the shape parameter is equal to 1, it simplifies to the exponential distribution. This adaptability makes the Weibull distribution a useful tool in both statistical analysis and engineering applications. While the shape parameter controls the tail behavior and thus affects skewness and kurtosis, the scale parameter adjusts the spread of the distribution without changing its overall shape.

In this study, we decided to generate samples from two Weibull distributions: one with a shape parameter less than 1 (shape = 0.5), indicating greater skewness, and another with a shape parameter greater than 1 (shape = 2), indicating moderate skewness. In both cases, the scale parameter was fixed at 1.

**Figure 4:**
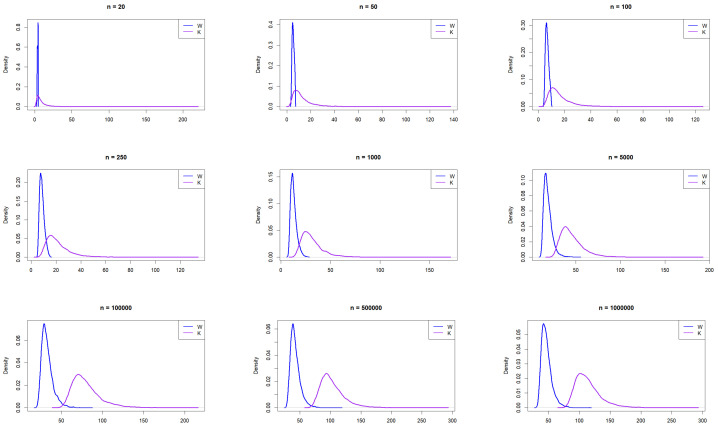
Empirical exploration of the distributions of *W* and *K* for n= 20, 50, 100, 250, 1000, 100,000, 500,000, and 1,000,000 from the Weibull distribution with shape = 0.5.

Figure 4 presents the empirical PDFs for *W* and *K* sampled from the Weibull distribution with shape = 0.5. Looking at the figure, it can be seen that K consistently exhibits a much more pronounced right skewness than W. Its density curves have long, heavy tails stretching toward larger values, and they are particularly visible, even as the sample size increases. In contrast, W is more concentrated, with a sharper peak and lighter tail, indicating less variability and skewness. This contrast suggests that K is more sensitive to extreme values or outliers, while W maintains a more compact distribution as the sample size increases.

**Figure 5:**
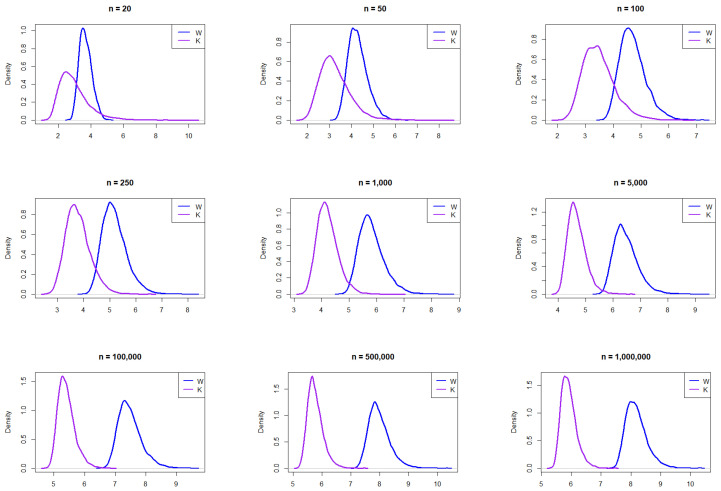
Empirical exploration of the distributions of *W* and *K* for n= 20, 50, 100, 250, 1000, 100,000, 500,000, and 1,000,000 from the Weibull distribution with shape = 2.

Figure 5 presents the empirical PDFs for *W* and *K* sampled from the Weibull distribution. Both have comparable shapes. Moreover, we can see that the overlap decreases as the sample size increases, suggesting that sample size plays a crucial role in determining their behavior.

## 3. Experimental Result

With the empirical distributions of *W* and *K* constructed in the preceding section, it is evident that large values of these two non-negative statistics indicate the potential presence of outliers. In this section, we present a comparison of the performance of *W* and *K* in detecting outliers based on the upper 1 percent, 5 percent, and 10 percent tail thresholds defining the outlier region. Table 1 gives the threshold values of the two range-based statistics, *W* and *K*, for each type of data, with a varying sample size (n=20 to n=1000) and level of error α (α=0.01,α=0.05,α=0.1). α represents the probability that at least one or more observations are wrongly declared outliers in the entire random sample of *n* regular observations. Smaller α values imply that very extreme data points would be flagged as outliers.

A quick examination of Table 1 reveals that, for a smaller sample size, *K* tends to produce higher threshold values than *W*, making it more conservative and less likely to classify data points as outliers unless their deviation is substantial. As the sample size increases, the thresholds of both statistics increase, reflecting the greater variability expected in larger datasets. For instance, the threshold for n=20 is significantly lower than that for n=500, as smaller datasets are less likely to exhibit extreme variations.

The authors in [14] developed an algorithm to examine the performance of the range statistics W and K in detecting outliers in a skewed distribution with a single error rate of 5 percent. Algorithm 1 is generalized here for any distribution and error rate (α) as follows:
**Algorithm 1:** General Outlier Detection Algorithm for Any Distribution
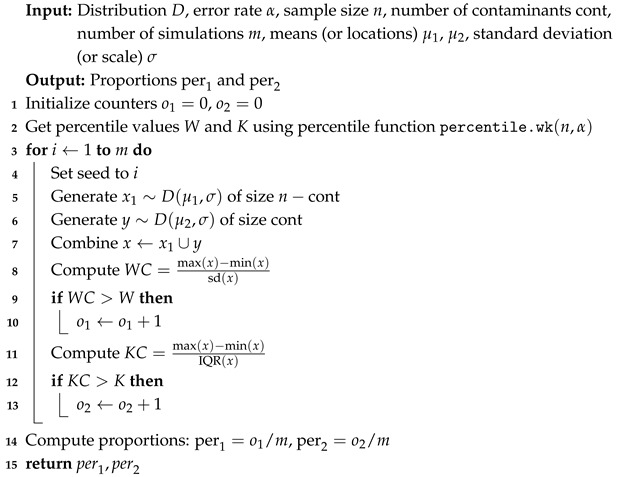


**Figure 6:**
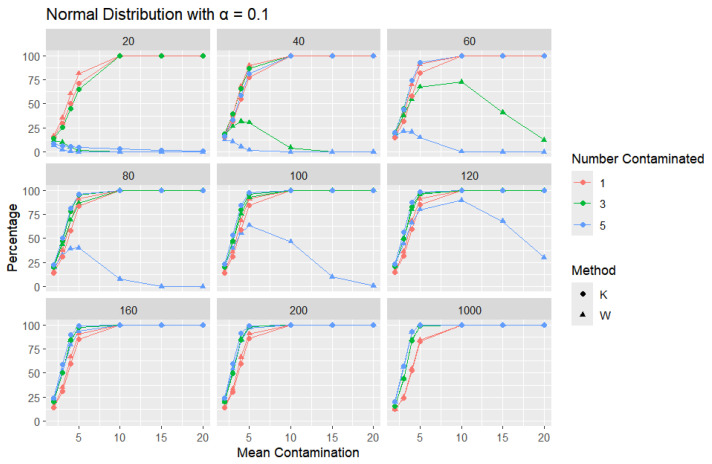
Line graphs of a 10% error rate for the percentage of detecting at least one outlier for the selected mean contamination values (x-axis). The bullet point line represents K percentages, and the triangle point line represents W percentages.

**Figure 7:**
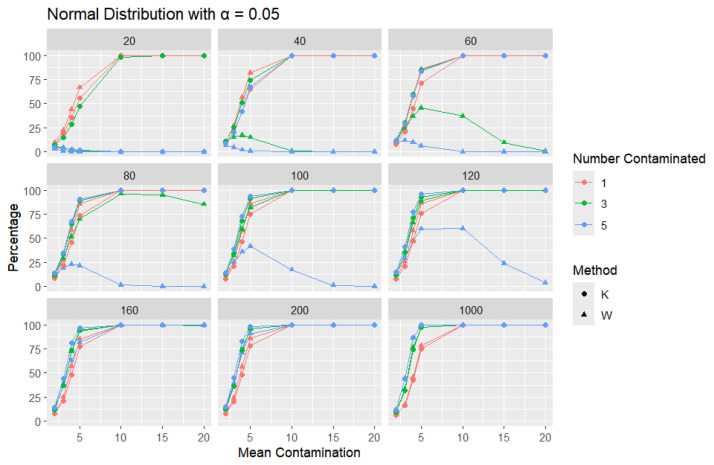
Line graphs of a 5% error rate for the percentage of detecting at least one outlier for the selected mean contamination values (x-axis). The bullet point line represents K percentages, and the triangle point line represents W percentages.

**Figure 8:**
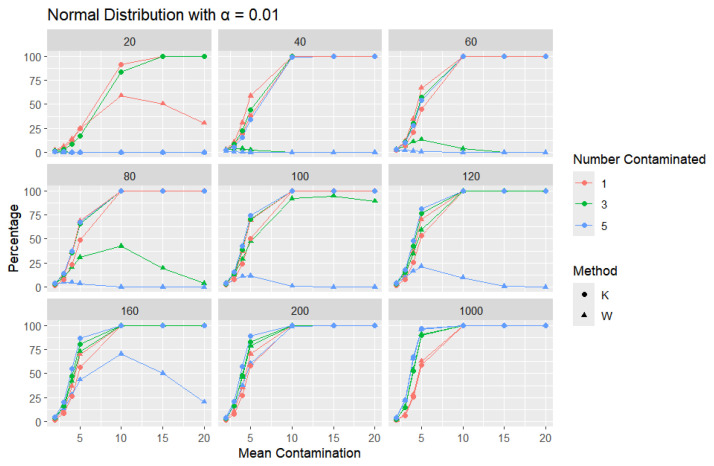
Line graphs of a 1% error rate for the percentage of detecting at least one outlier for the selected mean contamination values (x-axis). The bullet point line represents K percentages, and the triangle point line represents W percentages.

**Normal Distribution:** For each sample size, *n*, we contaminate each sample originally generated from the standard normal distribution (μ = 0, scale = 1) with 1, 3, and 5 points and different means (μ = 2, 3, 4, 5, 10, 15, and 20). The algorithm is applied, and the relative frequency of detecting at least one outlier is calculated for each sample size and each mean contamination. The results are plotted in Figure 6, Figure 7 and Figure 8.

The x-axis of each of the figures represents the selected contamination values of the location parameter, μ, and the y-axis represents the percentage of detecting at least one outlier. It is expected that, when contamination produces a distribution that deviates significantly from the original data distribution, the likelihood of outliers existing in the dataset increases, making the identification of such outlier values easier.

The results clearly show that *K* consistently outperforms *W* in detecting outliers. This disparity can be illustrated with a specific example: when looking at a sample size of 20, the contamination of five data points constitutes a significant portion, amounting to 25% of the data. As a result, these five points become indistinguishable from the rest of the dataset and are no longer flagged as outliers, even though they originate from a different mean. This leads *W* to detect a low percentage of outliers. The *W* statistic becomes highly influenced by the existence of unusual values in the data because the standard deviation is inflated in value by the unusual or outlier values. This trend is observable throughout but diminishes as the sample size increases. For large sample sizes, the statistics exhibit comparable behavior.

Moreover, *K*’s robustness is particularly evident at a smaller error rate (α=0.01), where *K* remains more reliable, while *W* struggles. This is a distinct advantage of *K*, especially that a lower error rate (α=0.01) produces a lower percentage than α=0.05 or α=0.1, particularly for small sample sizes and small mean contamination. For lower means (e.g., mean = 2), the detection percentages are generally low, as contamination may not deviate significantly from the central tendency. These findings highlight *K*’s superior ability to detect outliers across a range of conditions, making it a more effective choice.

**Figure 9:**
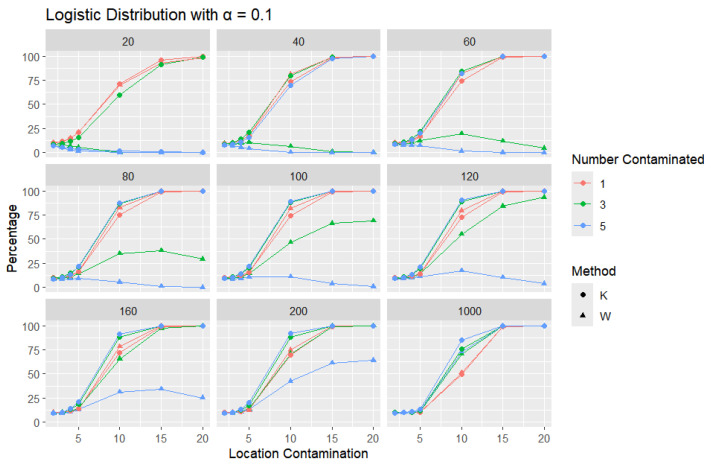
Line graphs of a 10% error rate for the percentage of detecting at least one outlier for the selected location contamination values (x-axis). The bullet point line represents K percentages, and the triangle point line represents W percentages.

**Figure 10:**
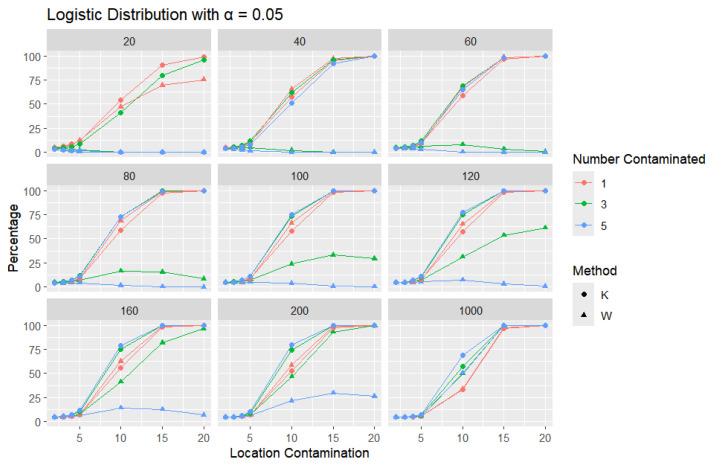
Line graphs of a 5% error rate for the percentage of detecting at least one outlier for the selected location contamination values (x-axis). The bullet point line represents K percentages, and the triangle point line represents W percentages.

**Figure 11:**
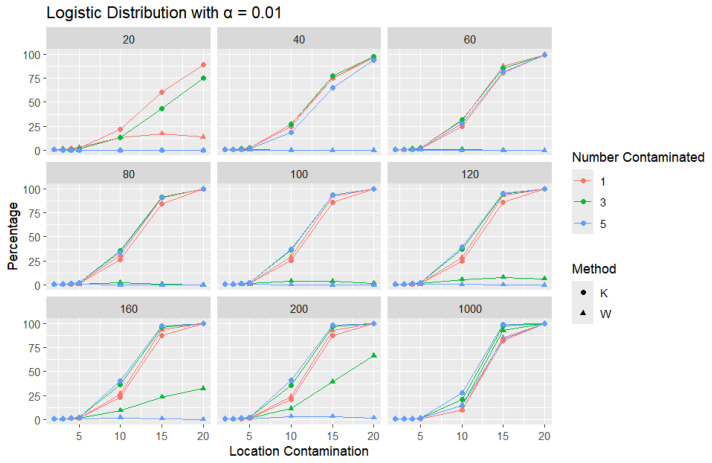
Line graphs of a 1% error rate for the percentage of detecting at least one outlier for the selected location contamination values (x-axis). The bullet point line represents K percentages, and the triangle point line represents W percentages.

**Logistic Distribution:** For each sample size, *n*, we contaminate each sample originally generated from the logistic distribution (μ = 0, scale = 1), with 1, 3, and 5 points and different locations (μ= 2, 3, 4, 5, 10, 15, and 20). The relative frequency of detecting at least one outlier is calculated for each sample size and each location contamination. The results are presented in Figure 9, Figure 10 and Figure 11.

In all scenarios, *K* consistently detects a higher percentage of outliers than *W*, particularly as the level of contamination and sample size increase. The difference in detecting outliers becomes more evident for higher contamination levels and lower values of α. Once again, this trend suggests that *K* is more sensitive to outliers, making it a preferable approach over *W*. It is important to note that the logistic distribution is considered a heavy-tailed distribution, meaning that its tails extend farther on both sides compared to those of the normal distribution. This characteristic is reflected in the high threshold values reported in Table 1. The figures show that, for smaller contamination values, the detection rate is very low (almost zero) for both statistics. However, at large contamination values, that is, 15 and 20, as the contamination value increases substantially, *K* outperforms *W* at small sample sizes, after which their performance becomes comparable.

**Figure 12:**
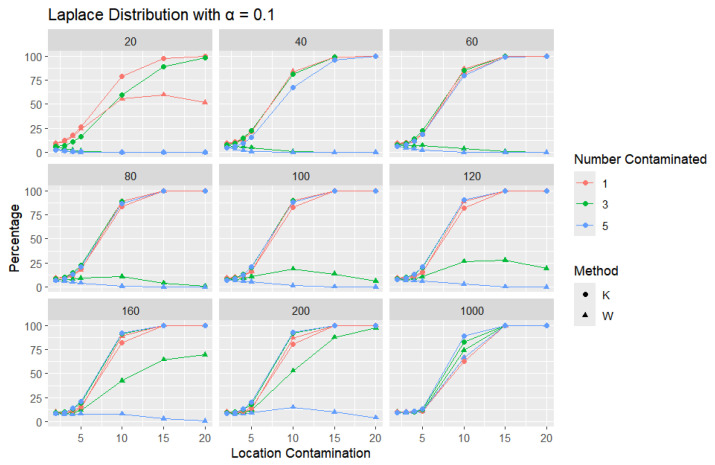
Line graphs of a 10% error rate for the percentage of detecting at least one outlier for the selected location contamination values (x-axis). The bullet point line represents K percentages, and the triangle point line represents W percentages.

**Figure 13:**
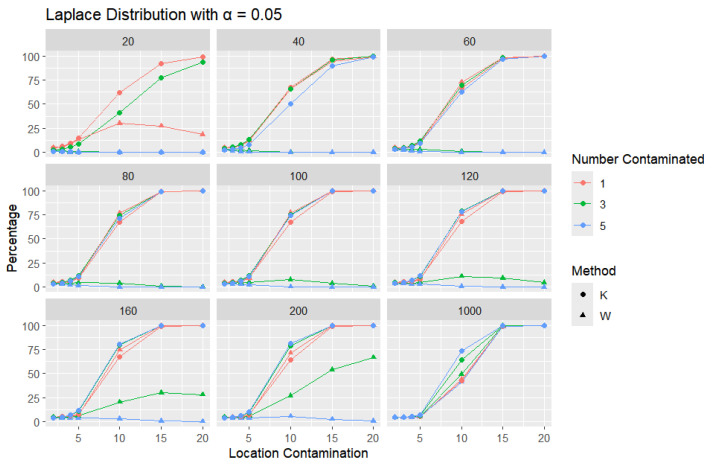
Line graphs of a 5% error rate for the percentage of detecting at least one outlier for the selected location contamination values (x-axis). The bullet point line represents K percentages, and the triangle point line represents W percentages.

**Figure 14:**
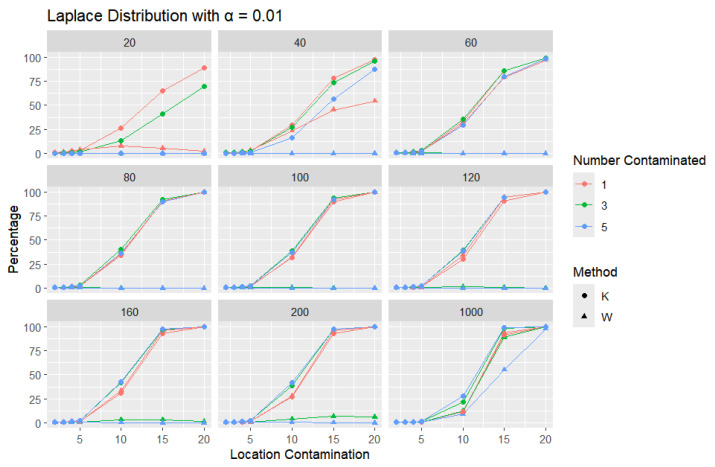
Line graphs of a 1% error rate for the percentage of detecting at least one outlier for the selected location contamination values (x-axis). The bullet point line represents K percentages, and the triangle point line represents W percentages.

**Laplace Distribution:** For each sample size, *n*, we contaminate each generated sample with 1, 3, and 5 points from a Laplace distribution with different locations (μ = 2, 3, 4, 5, 10, 15, and 20). The relative frequency of detecting at least one outlier is calculated for each sample size and each location contamination. The results are presented in Figure 12, Figure 13 and Figure 14.

A trend similar to that observed in the normal distribution can be seen in the figures for the Laplace distribution, where *K* outperforms *W*. However, the overall detection percentages are generally lower than those for the normal distribution. Specifically, with a smaller error rate (α=0.01), we see that the percentage of detecting at least one outlier is much lower than with a higher error rate (α=0.1), though *K* still maintains better performance than *W*. The Laplace distribution, just like the logistic distribution, is a heavy-tailed distribution, as reflected in the high threshold values shown in Table 1. This heavy-tailed nature makes outlier detection more challenging, especially when contaminating with small location shifts. The figures reveal that, for smaller contamination values, the detection rate is almost zero for both statistics. At higher mean values, such as 15 and 20, the detection rate increases, with *K* outperforming *W* with a smaller sample size. Therefore, *K* is more accurate in detecting outliers than *W* (has a high level of accuracy), showing its flexibility in terms of adjusting the detection method to the distribution of the data.

**Figure 15:**
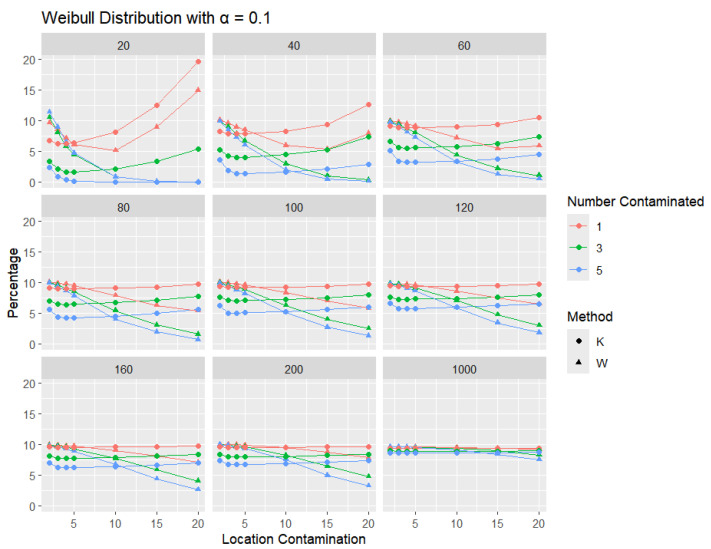
Line graphs of a 10% error rate for the percentage of detecting at least one outlier for the selected location contamination values (x-axis) from Weibull shape = 0.5. The bullet point line represents K percentages, and the triangle point line represents W percentages.

**Figure 16:**
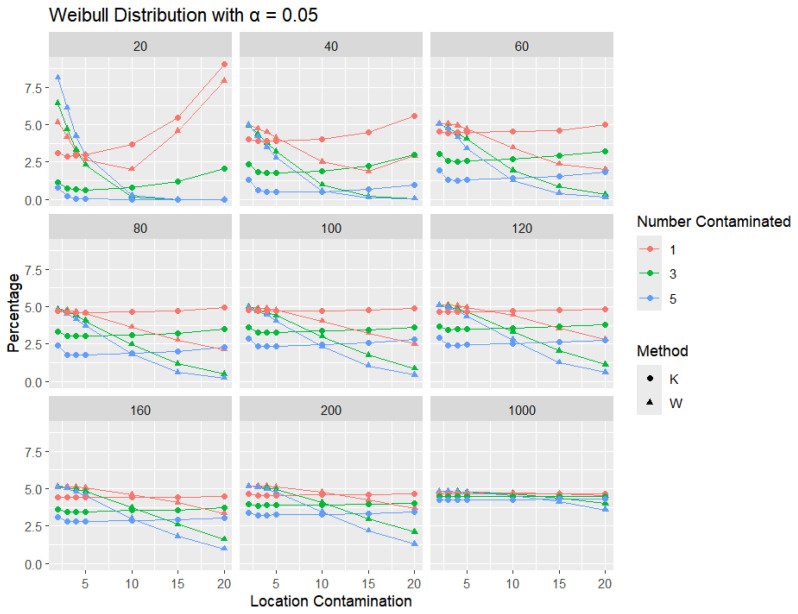
Line graphs of a 5% error rate for the percentage of detecting at least one outlier for the selected location contamination values (x-axis) from Weibull shape = 0.5. The bullet point line represents K percentages, and the triangle point line represents W percentages.

**Figure 17:**
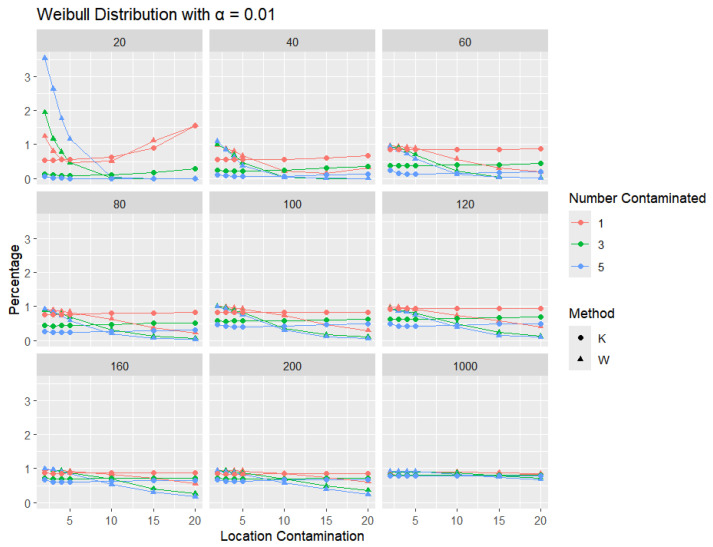
Line graphs of a 1% error rate for the percentage of detecting at least one outlier for the selected location contamination values (x-axis) from Weibull shape = 0.5. The bullet point line represents K percentages, and the triangle point line represents W percentages.

**Weibull Distribution:** For each sample size, *n*, we contaminate each generated sample with 1, 3, and 5 points from two different shape parameters of a Weibull distribution (shape = 0.5 and shape = 2) shifted by different locations (μ = 2, 3, 4, 5, 10, 15, and 20). The relative frequency of detecting at least one outlier is calculated for each sample size and each mean contamination. The results for a shape parameter of 0.5 are presented in Figure 15, Figure 16 and Figure 17.

Across all sample sizes, *K* consistently shows higher detection rates than *W*, particularly at smaller sample sizes and lower contamination levels. As the number of contaminated points increases, both methods tend to detect outliers more frequently; however, the relative advantage of *K* over *W* remains evident. As expected, with a stricter significance level, the detection rates of both methods are generally lower than in the 5% case. Nonetheless, *K* consistently demonstrates slightly higher detection percentages than *W*, especially at small sample sizes and when the contamination is mild. The performance gap gradually narrows with an increasing sample size, and both methods converge toward similar detection rates when the sample size reaches 1000. These results illustrate the robustness and sensitivity of *K*, especially under strong right-skewness and small-sample conditions.

**Figure 18:**
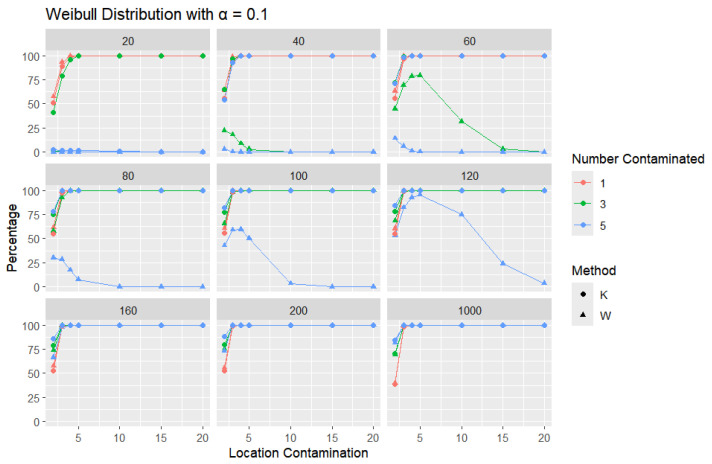
Line graphs of a 10% error rate for the percentage of detecting at least one outlier for the selected location contamination values (x-axis) from Weibull shape = 2. The bullet point line represents K percentages, and the triangle point line represents W percentages.

**Figure 19:**
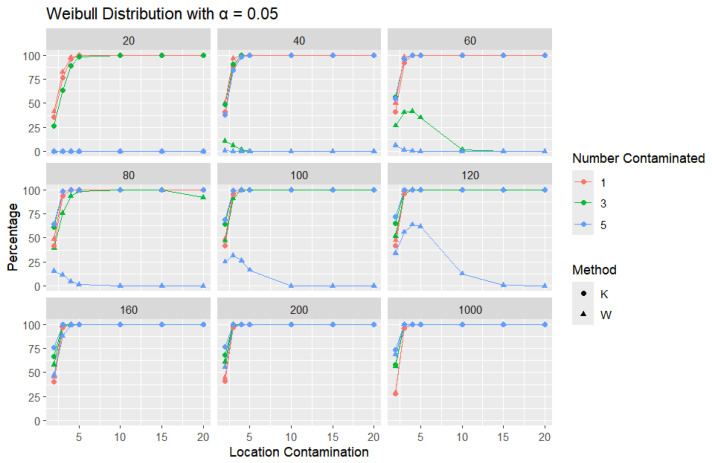
Line graphs of a 5% error rate for the percentage of detecting at least one outlier for the selected location contamination values (x-axis) from Weibull shape = 2. The bullet point line represents K percentages, and the triangle point line represents W percentages.

**Figure 20:**
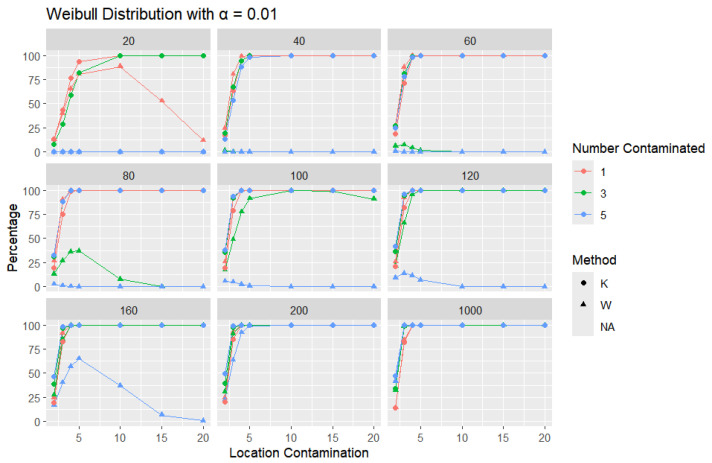
Line graphs of a 1% error rate for the percentage of detecting at least one outlier for the selected location contamination values (x-axis) from Weibull shape = 2. The bullet point line represents K percentages, and the triangle point line represents W percentages.

The results for a shape parameter of 2 are presented in Figure 18, Figure 19 and Figure 20.

Across all error rates and sample sizes, the *K* statistic consistently outperforms *W* in detecting outliers in the Weibull distribution. Notably, in the Weibull case, both *K* and *W* generally achieve much higher detection rates than in the normal, logistic, and Laplace distributions. This can be attributed to the skewed nature of the Weibull distribution, where the asymmetry makes outliers more distinguishable from the rest of the data. For small sample sizes (n=20,40,60), *K* detects outliers at a very high rate, even with small increases in location contamination, while the performance of *W* remains extremely low, often close to zero. As the sample size increases (n≥100), both *K* and *W* detect outliers more effectively, but *K* still identifies them more quickly and consistently, especially at lower contamination levels.

## 4. Discussion

The experimental results in this study provide important insights into the performance of the standardized range statistic *W* and the relative range statistic *K* in detecting outliers across different probability distributions. While both statistics aim to identify anomalous observations in datasets, an analysis revealed clear distinctions in their behavior and effectiveness depending on the underlying distribution, sample size, contamination level, and error rate. Empirical analyses across four distributions, namely, normal, logistic, Laplace, and Weibull distributions, consistently highlighted the relative range statistic *K* as a more robust outlier detection method than the standardized range *W*, particularly in skewed distributions and under small error rates.

For the **normal distribution**, *K* consistently detected a higher proportion of outliers across sample sizes and contamination levels, particularly at a lower error rate of α=0.01. This advantage became more prominent as the contamination mean increased, demonstrating *K*’s superiority to subtle anomalies in normally distributed data. Interestingly, for larger samples (e.g., n≥100), both methods showed convergence in performance, which might explain why earlier works, such as that by Harter [11], focused only on sample sizes up to 100, where the sensitivity and variability of range-based statistics are the most noticeable. In the **logistic and Laplace distributions**, which are symmetric but have heavier tails than the normal distribution, *K* clearly outperformed *W* in almost all scenarios. Both distributions exhibited low detection rates, particularly at smaller contamination levels and lower error rates. This was expected, as heavy tails tend to hide moderate outliers. The advantage was particularly evident for smaller sample sizes and higher location contamination values, where *W*’s detection rates increased only marginally, while *K* continued to show improved performance. Interestingly, even when the error rate decreased or the sample size increased, *K* kept performing better, while *W* stayed small, with an insignificant increase in the percentage. This shows that *K* handles data spread and variability more effectively. The most remarkable results emerged from the **Weibull distribution** with shape = 2, which is positively skewed. The detection rates for both statistics were significantly higher than in the other distributions. Even for small contamination values and small samples, *K* achieved an almost 100% detection rate in many scenarios. This suggests that skewed distributions may inherently increase the visibility of outliers, making range-based methods more effective.

**Figure A1:**
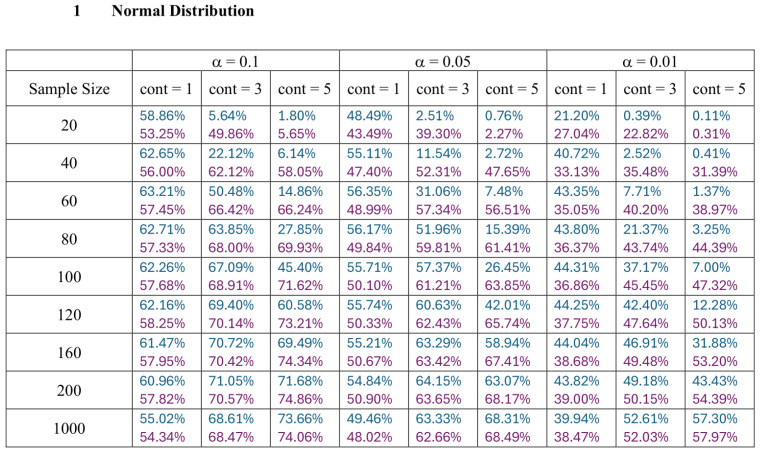
Average percentages of W and K for each number of contaminated points and each sample size from the normal distribution: blue indicates the average percentage of W, and purple indicates the average percentage of K.

**Figure A2:**
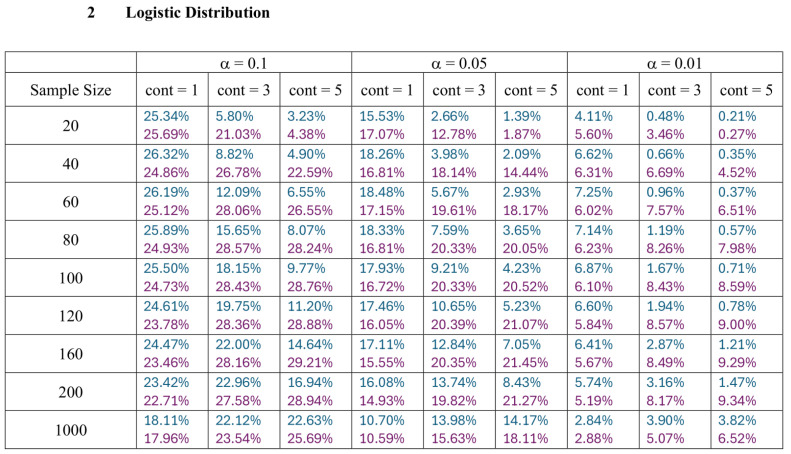
Average percentages of W and K for each number of contaminated points and each sample size from the logistic distribution: blue indicates the average percentage of W, and purple indicates the average percentage of K.

**Figure A3:**
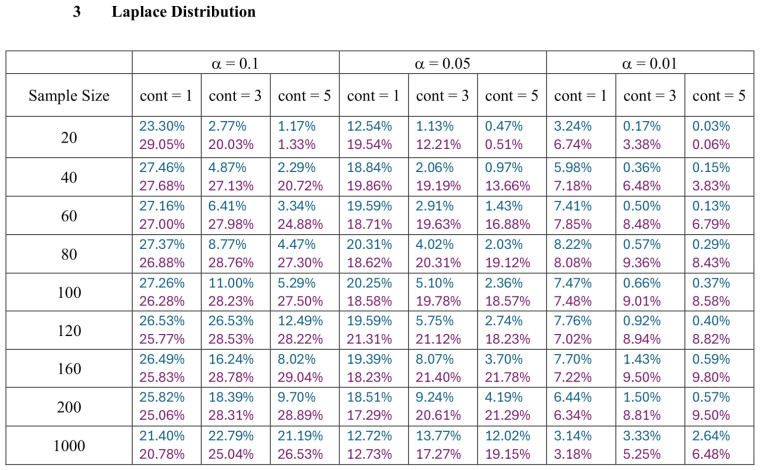
Average percentages of W and K for each number of contaminated points and each sample size from the Laplace distribution: blue indicates the average percentage of W, and purple indicates the average percentage of K.

**Figure A4:**
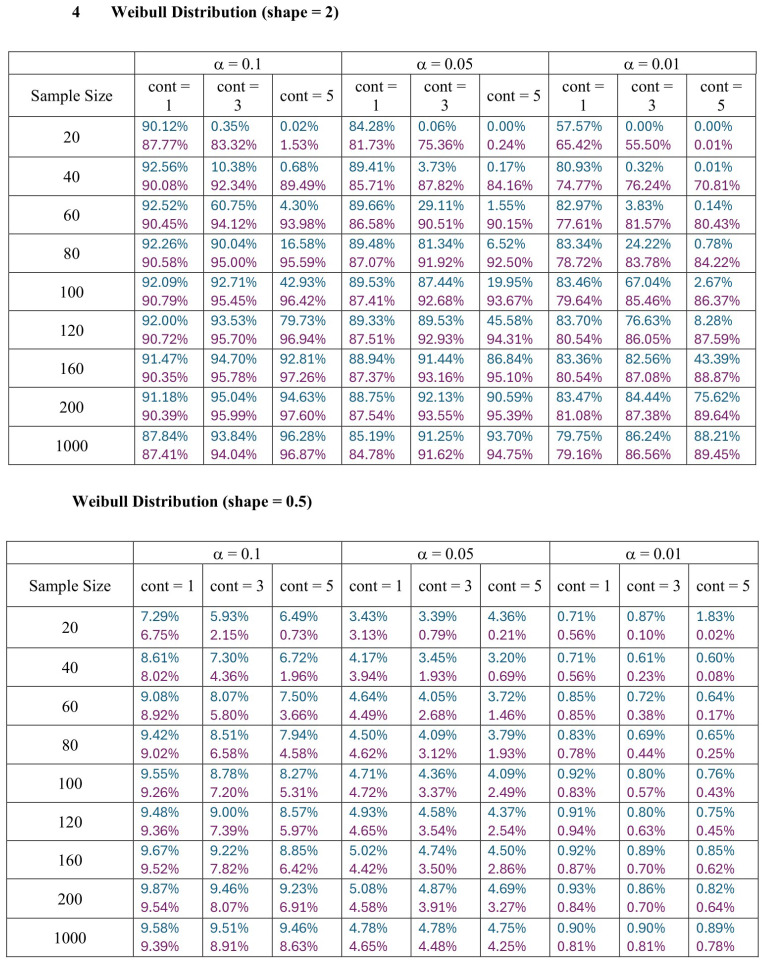
Average percentage of W and K for each number of contaminated points and each sample size from the Weibull distribution with shape parameters of 0.5 and 2: blue indicates the average percentage of W, and purple indicates the average percentage of K.

In terms of performance across multiple error rates (α = 0.1, 0.05, 0.01), *K* appeared to maintain a more stable behavior than *W*, further establishing its robustness under various testing conditions. Lower α values were associated with lower detection rates for both K and W. An important advantage of our simulation-based approach is its reliance on the empirical probability characteristics of the data. This is particularly useful when working with real-world datasets where the underlying distribution is unknown or deviates from normality. By leveraging the distributional behavior of *K*, this method provides a flexible and adaptable framework for practical outlier detection. In general, the *K* statistic proves to be a more flexible and reliable method, especially when working with data that are not normally distributed. *K* is robust in terms of maintaining accuracy in outlier detection under various conditions. It adapts better across different distributions and sample sizes, making it a solid choice for feasible outlier detection. This is also reflected in the numerical summaries of the line graphs, presented as the average percentage detected for each distribution, which can be found in the Appendix Figure A1, Figure A2, Figure A3 and Figure A4. In addition to its statistical performance, the relative range statistic *K* is computationally efficient and straightforward to implement. It requires only the calculation of the sample maximum, minimum, and quartiles.

### Analytical Justification of K’s Performance

The use of the IQR to standardize the range statistic plays a critical role in enhancing the performance and robustness of *K* for outlier detection. Unlike the standard deviation, which is highly sensitive to extreme values, the IQR is a robust measure of scale that focuses on the middle 50% of the data and is therefore resistant to distortion by outliers. When the standard deviation is used, a single extreme observation can inflate the estimate of variability, masking the presence of additional outliers and leading to inconsistent performance. In contrast, dividing by the IQR stabilizes the behavior of the statistic across different distributional shapes and tail behaviors. This robustness is particularly important when dealing with skewed or heavy-tailed distributions, where the presence of extreme values is more common. As a result, *K* offers a more consistent detection of outliers across a wide range of settings, which is supported by the empirical findings presented in this study.

## 5. Conclusions

This study evaluated the effectiveness of two range-based statistics, the standardized range *W* and the relative range *K*, for outlier detection across four common distributions and a variety of sample sizes. By normalizing the range by the interquartile range, *K* proved to be better than *W* at handling outlier values in different data scenarios. Across all distributions, *K* showed higher detection rates than *W*, with the difference being more noticeable in heavy-tailed or skewed distributions like Laplace and Weibull distributions. In the Weibull case, both methods performed better than in the other distributions, but *K* still produced higher and more stable detection rates than *W*. These results consistently remained true for different error rates (α=0.1,0.05,0.01) and varying numbers of contaminants. Consequently, the findings confirm that *K* is a more effective and reliable tool for detecting outliers, particularly for small sample sizes and non-normally distributed datasets.

It would be of interest for future work to see how *K* performs in more complex situations such as multivariate data or real-world applications where the underlying distribution is unknown. But for univariate outlier detection, especially in exploratory analysis, *K* appears to be a strong contender.

## Figures and Tables

**Table 1:** The 99th, 95th, and 90th percentiles of W and K from Normal, Logistic, Laplace and Weibull Distributions.

Sample Size	Normal						Logistic						Laplace						Weibull (Shape = 2)						Weibull (Shape = 0.5)					
	α=0.01		α=0.05		α=0.1		α=0.01		α=0.05		α=0.1		α=0.01		α=0.05		α=0.1		α=0.01		α=0.05		α=0.1		α=0.01		α=0.05		α=0.1	
	*W*	*K*	*W*	*K*	*W*	*K*	*W*	*K*	*W*	*K*	*W*	*K*	*W*	*K*	*W*	*K*	*W*	*K*	*W*	*K*	*W*	*K*	*W*	*K*	*W*	*K*	*W*	*K*	*W*	*K*
n=20	4.807	6.341	5.336	4.934	4.321	4.376	5.075	7.910	4.768	5.996	5.920	5.251	5.336	11.233	5.027	8.242	4.849	7.092	4.639	6.005	4.355	4.694	4.187	4.155	4.569	47.569	4.525	25.573	4.476	18.865
n=30	5.271	5.981	6.166	4.872	4.703	4.397	5.767	7.670	5.350	6.112	6.308	5.920	6.166	11.121	5.726	8.519	5.521	7.508	5.047	5.670	4.691	4.567	4.511	4.131	5.5185	48.705	5.430	27.395	5.318	20.915
n=50	5.765	5.663	7.228	4.842	5.143	4.468	6.614	7.577	6.075	6.278	5.792	5.696	7.228	11.114	6.649	8.915	6.365	7.990	5.532	5.410	5.098	4.492	4.885	4.144	7.005	48.232	6.751	30.897	6.507	23.942
n=75	6.119	5.549	8.103	4.872	5.463	4.552	7.608	7.650	6.623	6.455	5.124	5.445	8.103	11.185	7.379	9.245	7.029	8.377	5.860	5.191	5.389	4.512	5.154	4.187	8.401	50.318	7.957	32.998	7.584	26.285
n=100	6.338	5.543	5.900	4.914	5.676	4.622	7.750	7.747	7.028	6.635	6.676	6.115	8.703	11.340	7.870	9.560	7.500	8.696	6.038	5.184	5.564	4.534	5.335	4.239	9.541	51.987	8.897	34.821	8.397	27.666
n=250	6.996	5.600	6.533	5.121	6.305	4.889	9.119	8.247	8.235	7.282	7.818	6.810	10.527	12.141	9.487	10.568	8.979	9.809	6.556	5.084	6.054	4.630	5.809	4.412	13.837	56.954	12.335	40.538	11.3701	33.946
n=500	7.423	5.732	6.953	5.318	6.725	5.117	10.064	8.776	9.107	7.820	8.662	7.378	11.783	12.957	10.637	11.380	10.077	10.708	6.909	5.195	6.386	4.764	6.133	4.551	17.802	64.879	15.402	46.541	13.990	39.316
n=1000	7.787	5.912	7.337	5.540	7.117	5.350	10.939	9.321	9.927	8.363	9.477	7.968	12.948	13.815	11.695	12.274	11.137	11.640	7.148	5.287	6.666	4.900	6.432	4.712	22.115	70.792	18.799	53.101	16.911	45.832

## Data Availability

Data are contained within the article.

## Appendix A

Numerical descriptions of the line graphs are represented in the tables below as the average percentages of each distribution.
